# *Acacia nilotica* Pods’ Extract Assisted-Hydrothermal Synthesis and Characterization of ZnO-CuO Nanocomposites

**DOI:** 10.3390/ma15062291

**Published:** 2022-03-20

**Authors:** Manal Hessien, Amel Taha, Enshirah Da’na

**Affiliations:** 1 Department of Chemistry, College of Science, King Faisal University, P.O. Box 400, Al-Ahsa 31982, Saudi Arabia; ataha@kfu.edu.sa; 2 Department of Chemistry, Faculty of Science and Technology, Al-Neelain University, Khartoum 11121, Sudan; 3 Department of Biomedical Engineering, Faculty of Engineering, King Faisal University, P.O. Box 400, Al-Ahsa 31982, Saudi Arabia; edana@kfu.edu.sa

**Keywords:** *Acacia nilotica*, wurtzite ZnO, tenorite CuO, nanocomposite, microstructure

## Abstract

This work represents a novel combination between *Acacia nilotica* pods’ extract and the hydrothermal method to prepare nanoparticles of pure zinc oxide and pure copper oxide and nanocomposites of both oxides in different ratios. Five samples were prepared with different ratios of zinc oxide and copper oxide; 100% ZnO (ZC0), 75% ZnO: 25% CuO (ZC25), 50% ZnO: 50% CuO (ZC50), 25% ZnO: 75% CuO (ZC75), and 100% CuO (ZC100). Several techniques have been applied to characterize the prepared powders as FTIR, XRD, SEM, and TEM. The XRD results confirm the formation of the hexagonal wurtzite phase of zinc oxide and the monoclinic tenorite phase of copper oxide. The microscopy results show the formation of a heterostructure of nanocomposites with an average particle size of 13–27 nm.

## 1. Introduction

Zinc oxide has been widely applied as photocatalysts [1,2,3,4], antimicrobial agents [5,6,7], supercapacitors [8,9], sensors [6,10], varistor [11], biosensors [10], in solar cells [12,13,14], transparent electrodes [15], and fuel cells [9,16]. This wide range of applications is based on the characteristics of ZnO as an n-type semiconductor, low-cost, chemical, and thermal stability, a direct band gap (~3.3 eV), and binding energy of 60 meV [17,18]. Despite all these characteristics, there are two limitations to commercializing zinc oxide applications. First, its light absorption falls in the UV region, which deprived zinc oxide of the visible light absorption. Second, its high rate of charge recombination collapses its photocatalytic activity. To overcome these drawbacks, a doping zinc oxide or forming composite is applied [6].

Q. Zeng et al. reported enhanced gas sensing properties of NiO-ZnO nanocomposites, which they attributed to the formation of a p-n heterojunction [19]. The same observation was reported by the Yang group regarding the fast response of the Co_3_O_4_-ZnO gas sensor towards triethylamine [20]. Y. Wang et al. successfully enhanced the absorption of zinc oxide to be in the visible region through forming ZnO-MnO with a bandgap = 2.2 eV with Mn^2+^ = 50 at.% [21]. M. Toe et al. tuned the bandgap of ZnO through compositing it with CuO, NiO, and Al_2_O_3_, which changed the bandgap to 3.17 eV, 3.28 eV and 3.16 eV, respectively [22]. Copper oxide is a p-type semiconductor with a bandgap in the range of 1.2–2 eV. CuO is characterized by a low-cost and non-toxic material which makes it a good component to form a composite with ZnO.

Various methods are applied to prepare metal oxide nanoparticles and nanocomposites as simple precipitation [23], electrochemical method [24], mechanical milling [1], sol-gel method [2,25], biocombustion [26], co-precipitation [27], green synthesis [28,29], sonochemical synthesis [30] and hydrothermal method [31,32]. The hydrothermal method has many advantages as being simple, low-cost, and low-temperature. Green synthesis involves the use of plant extracts as capping agents and/or reducing agents instead of using synthetic surface directing agents as surfactants or polymers which may harm the environment [18,33,34].

R. Mohamed et al. prepared CuO-ZnO heterojunction photocatalyst through a simple wet method in the presence of F-127 surfactant [35]. A. Prajapati et al. prepared CuO-ZnO-C nanocomposites by using extract of *Tagetes* spp. petals in the presence of CTAB surfactant [36]. Moreover, the vapor deposition technique was used to prepare CuO-ZnO and applied in optoelectronics [37]. ZnO-CuO has been prepared by green synthesis using *Sambucus nigra* L. extract [28].

*Acacia nilotica*, also known as gum arabic tree, belongs to the *Fabaceae* family. It is popular in Africa and Asia and the tree has roots, pods, stems, and leaves. The extract has many phytochemical components as flavonoids, phenols, and tannins [38,39]. The pods have been applied in the synthesis of copper oxide nanoparticles [40], the leaves have been used in the preparation of silver nanoparticles [38], the aerial parts of *Acacia nilotica* have been used to prepare Ag-TiO_2_ nanocomposites [41].

The recent work reports for the first time the combination between a green synthesis that employs *Acacia nilotica* Pods’ extract and the hydrothermal method. Pristine zinc oxide and copper oxide nanoparticles were synthesized in addition to copper oxide-zinc oxide nanocomposites with different ratios. The functional groups and the formed phases were determined by Fourier Transform Infrared spectroscopy (FTIR) and X-ray diffraction (XRD), respectively. The morphology and particle size were followed by scanning electron microscopy (SEM) and transmission electron microscopy (TEM).

## 2. Materials and Methods

### 2.1. Materials and Instruments

Copper chloride hexahydrate (CuCl_2_.6H_2_O), zinc chloride (ZnCl_2_), potassium hydroxide (KOH) were bought from Sigma-Aldrich. All the primary chemicals used in this work were of analytical grade. All solutions were prepared with double-distilled water. The *Acacia nilotica* pods were bought from a local store, Al-Ahsa, Saudi Arabia.

The FTIR analysis of the copper oxide and zinc oxide nanoparticles and nanocomposites was analyzed on Cary 630 FT-IR spectrophotometer. The XRD analysis was performed on Bruker D8 X-ray Diffractometer with Ni-filtered Cu-Kα radiation and a graphite monochromator to produce X-rays with a wavelength of 1.5418 Å at 35 kV and 25 mA, using glancing-angle from 10° to 60° at scan steps of 0.02° with an accuracy ≤0.001°. For further surface morphology, Scanning Electron Microscope (SEM) (Philips XL30) was used, accelerating voltage of 30 kV and the magnification up to 400,000×. A high-resolution, JEOL JEM-1011 Transmission Electron Microscope was used for TEM imaging.

### 2.2. Acacia nilotica Extraction

The *Acacia nilotica* pods were rinsed with tap water three times and with distilled water for the last time. To prepare the extract, 10 g of dried pods were boiled with 100 mL of distilled water at 60 °C for 15 min under magnetic stirring. The prepared extract was centrifuged at 8000 rpm for 5 min and then filtered via Whatman No. 1 filter paper, to remove all fine plant debris and stored at 4 °C [42]. The pH of the extract is 5.71.

### 2.3. Green Synthesis

A 0.2 M solution of copper chloride hexahydrate (CuCl_2_.6H_2_O) and a 0.2 M solution of zinc chloride (ZnCl_2_) were prepared in two different containers. The compositions of samples are mentioned in Table 1. As an example, 20 mL of the *Acacia nilotica* extract is added to a 100 mL of 0.2 M zinc chloride salt solution to prepare sample ZC0. A 1 M KOH was added to the previous mixture while under continuous stirring until the pH reaches 10.

### 2.4. Hydrothermal Synthesis

**Scheme 1:**
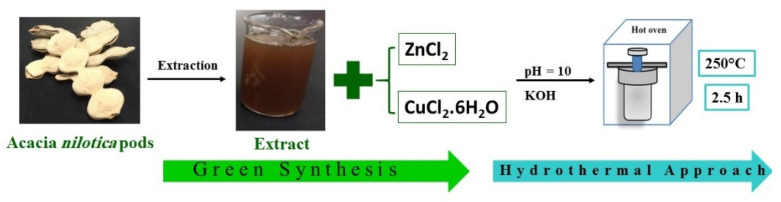
The synthesis steps for samples prepared by green assisted-hydrothermal method.

The prepared precipitate was transferred to a Teflon-lined autoclave, which was placed in an oven at 250 °C for 2.5 h. After the completion of hydrothermal treatment, the solution is subjected to sonication for an hour in 100 mL distilled water. The sample was then left to settle down and the liquor was disposed of. The sonication and settling down steps were repeated three times with water and one time with ethanol. The sonication was executed by using Power sonic405 ultrasonic bath to cleanse the samples. The samples were dried in an oven overnight at 75 °C. In the end, the samples were calcined at 300 °C for 2 h to get rid of the extract residuals. Scheme 1 represents the synthesis steps.

## 3. Results

### 3.1. FTIR Characterization

**Figure 1:**
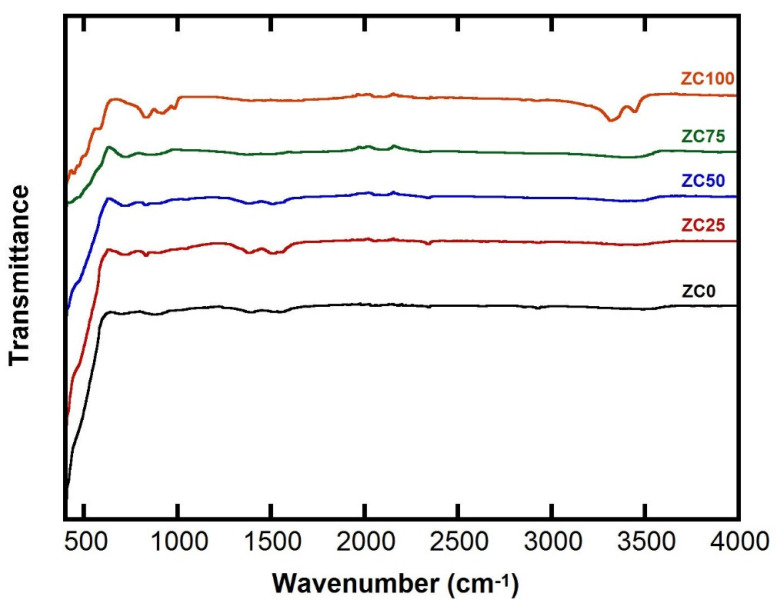
FTIR spectra of the ZC0, ZC25, ZC50, ZC75, and ZC100 samples.

Figure 1 represents the FTIR of ZC0, ZC25, ZC50, ZC75 and ZC100 samples after calcination at 300 °C for 2 h. In some samples, a shallow broad peak around 3200 cm^−1^ is assigned for the OH group. The peak at 1600 cm^−1^ is assigned for absorbed water molecules from the surrounding [43]. The peaks below 700 cm^−1^ can be assigned to metal–oxygen bonds in both zinc oxide and copper oxide [44]. In sample ZC0, which is pristine zinc oxide, the deep peak below 700 cm^−1^ is assigned for the Zn-O bond [32]. The depth of this peak decreases as the content of ZnO decreases in samples ZC25, ZC50 and ZC75, respectively. In sample ZC100, which is pristine copper oxide, the less deep peak below 600 cm^−1^ indicates the presence of Cu-O. Going from sample ZC75, ZC50 to ZC25, the depth of this band increases as the content of copper oxide decreases and the content of zinc oxide increases [26]. The FTIR results confirm the formation of pristine zinc oxide, zinc oxide-copper oxide nanocomposites and pristine copper oxide in samples ZC0, ZC25, ZC50, ZC75, and ZC100, respectively.

**Figure 2:**
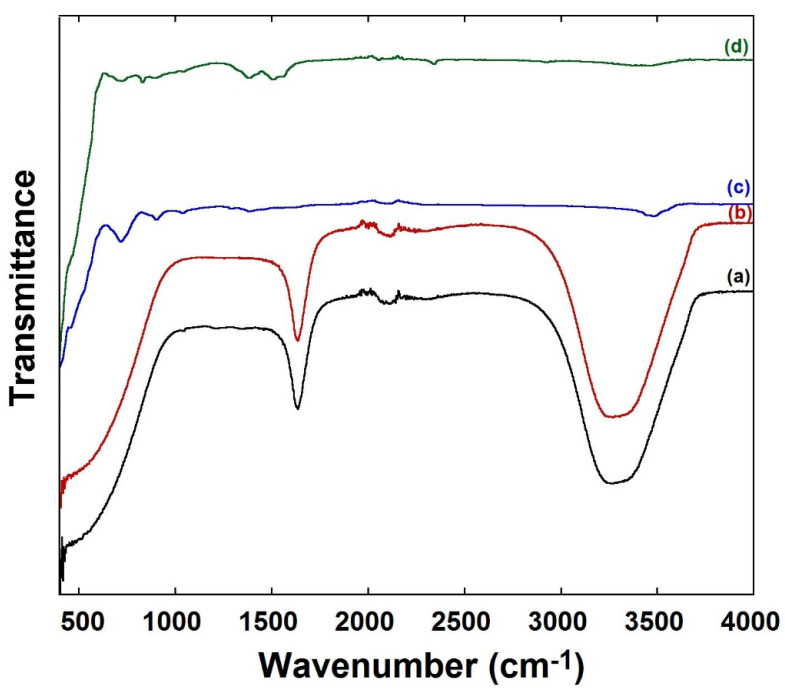
The FTIR spectra of (**a**) Acacia extract. (**b**) Sample ZC50 after precipitation. (**c**) Sample ZC50 after hydrothermal treatment and drying. (**d**) Sample ZC50 after calcination.

Figure 2a represents the FTIR of *Acacia nilotica* pods’ extract, sample ZC50 (b) after precipitation, (c) after hydrothermal treatment and drying and (d) after calcination. The FTIR of *Acacia nilotica* pods’ extract shows a very broad band between 2800–3600 cm^−1^ which may correspond to the stretching vibration of aromatic and aliphatic OH groups (3200–3500 cm^−1^) or stretching of aromatic and aliphatic C-H (3000–3100 cm^−1^). A sharp finger-like peak around 1500–1700 cm^−1^ stands for carbonyl groups or bending of aromatic C=C. A very broad band below 1000 cm^−1^ may refer to the bending of aromatic C-H or C-O-C groups. According to the literature, the FTIR results of *Acacia nilotica* pods’ extract confirm the presence of flavonoids, tannins and terpenoids in the extract [38,39]. The FTIR spectrum of sample ZC50 after adding KOH is shown in Figure 2b where all the peaks of the extract can be seen. Figure 2c shows the FTIR spectrum of the sample after the hydrothermal treatment and drying which results in the disappearance of the peaks related to the extract. Figure 2d shows the FTIR of the sample after calcination at 300 °C for 2 h, which results in the sharpness of peaks related to M-O bonds between 400–600 cm^−1^ [26].

### 3.2. XRD Characterization

**Figure 3:**
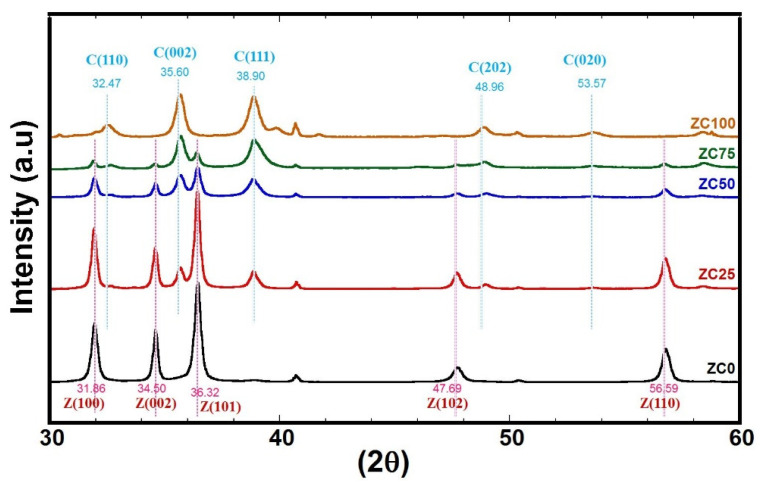
The XRD patterns of the ZC0, ZC25, ZC50, ZC75, and ZC100 samples.

Figure 3 represents the XRD patterns of the samples ZC0, ZC25, ZC50, ZC75, and ZC100 prepared by the green-hydrothermal technique (pH = 10, T = 250 ◦C, t = 2.5 h) and calcined at 300 °C for 2 h. The ZC0 sample shows peaks at 2θ~31.96°, 34.61°, 36.44°, 47.74°, and 56.73° corresponding to the diffraction planes (1 0 0), (0 0 2), (1 0 1), (0 1 2), and (1 1 0) of a wurtzite hexagonal structure according to Card no. 96-900-4179 [36]. The XRD results confirm that sample ZC0 is formed completely of zincite, which is pure zinc oxide with a hexagonal phase. For the sample ZC100, the peaks appear at 2θ~32.47°, 35.60°, 38.90°, 48.96°, and 53.576° for (1 1 0), (11-1), (1 1 1), (2 0-2), and (0 2 0) according to Card no. 96-721-2243, which represents the monoclinic phase of copper oxide. The XRD results confirm that sample ZC100 is formed completely of tenorite, which is pure copper oxide with a monoclinic phase [36,45,46,47].

Samples ZC25, ZC50, and ZC75 show the peaks corresponding to wurtzite hexagonal zinc oxide and tenorite monoclinic copper oxide with different intensities. However, the intensity of peaks corresponding to wurtzite zinc oxide is decreasing gradually in the samples ZC25, ZC50, and ZC75 with decreasing the amount of zinc precursor in the starting materials. The same can be observed in an opposite way for the monoclinic tenorite and the intensity of copper oxide peaks increases gradually in samples ZC25, ZC50, ZC75 with an increasing copper precursor in the initial compositions (Table 1) [2,26,48]. There are no peaks detected by XRD for Zn metal, Cu metal, or any other compounds which indicates the high purity of formed ZnO-CuO nanocomposites. In addition, there is no observed shift in the corresponding peaks of either ZnO or CuO, which indicates the heterostructure formation of ZnO-CuO and no incorporation [49].

**Figure 4:**
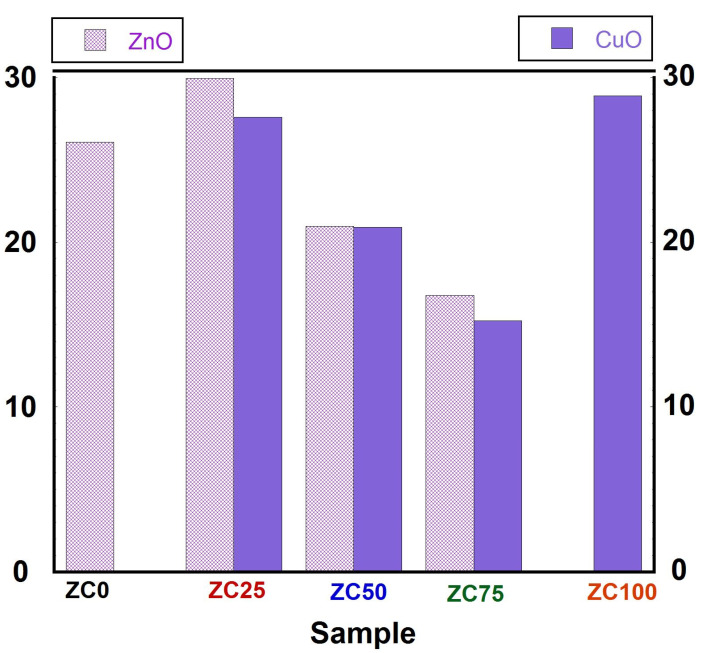
The crystallite size as calculated by Scherrer’s Equation of the ZC0, ZC25, ZC50, ZC75, and ZC100 samples.

The crystallite size of zinc oxide and copper oxide is estimated using the Debye–Scherrer formula and presented in Figure 4 [50]. The crystallite size of zinc oxide is calculated based on the main peak corresponding to plane (101) and based on the main peak corresponding to plane (111) of tenorite copper oxide. The crystallite size of pristine zinc oxide is 26 nm, and the crystallite size of pristine copper oxide is 29 nm. It seems that adding copper oxide (25%) to zinc oxide derives a rise in the crystallite size of zinc oxide to 30 nm compared to pristine zinc oxide (26 nm). Further increase of copper oxide (50% and 75%) develops a decrease in the crystallite size of zinc oxide to 21 nm and 17 nm, respectively. On the other hand, adding zinc oxide to copper oxide produces a decrease in the crystallite size of copper oxide to 15 nm, 21 nm, and 27 nm in samples (ZC 75, ZC50, and ZC25) compared to 29 nm of pristine copper oxide. A. Lavin et al. prepared ZnO-CuO nanocomposites through the sol-gel method and found that the crystallite size of zinc oxide and copper oxide varied with composition [47]. It is worth noting that in their results when a sample has equal amounts of zinc oxide and copper oxide; the crystallite size is about 48 nm for both oxides. In another study, the average crystallite size of ZnO/CuO nanocomposite powders was 30 nm when the solvent was water and it decreased to 25 nm when the solvent changed to water/ethanol [44]. While in this work, sample ZC50 has a crystallite size of 21 nm for both oxides. This may be related to the effect of capping agents found in *Acacia nilotica* extract, which seems to be more effective as a capping agent than ethanol as a solvent in decreasing the crystallite size [47]. C. Kumar et al. prepared ZnO-CuO by combustion method assisted with extract of *Calotropis gigantea*, and the average crystallite size was about 35 nm [29]. M. Mansourina et al. reported that the average crystallite size of hydrothermally synthesized ZnO and CuO are 26 and 21 nm, respectively. It is worth noting that the mentioned crystallite size is for the as-prepared samples, which are smaller than the crystallite size of the calcined samples [51]. Again, the integration of hydrothermal with green synthesis resulted in a less average crystallite size. Ultimately, the XRD results show that the green assisted-hydrothermal method successfully synthesizes zinc oxide nanoparticles, copper oxide nanoparticles, and zinc oxide–copper oxide nanocomposites with no other alloys or intermediate compounds with crystallite size 15–29 nm.

### 3.3. Morphology

**Figure 5:**
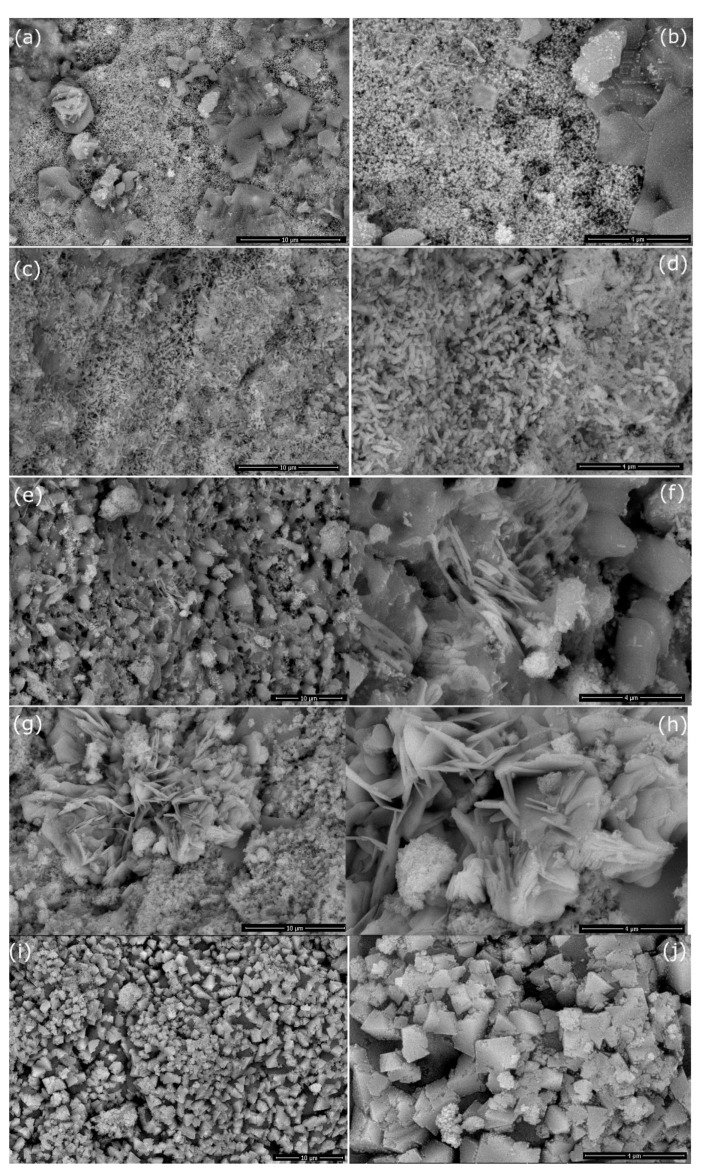
The SEM photos of the ZC0 (**a**,**b**), ZC25 (**c**,**d**), ZC50 (**e**,**f**), ZC75 (**g**,**h**), and ZC100 (**i**,**j**) samples.

Figure 5 represents the surface morphology of the different samples as investigated by SEM. Sample ZC0 is shown in Figure 5a,b and its morphology is composed of agglomerated particles along with stacked layers. In our previous work on hydrothermally prepared pristine ZnO without capping agents, ZnO had a morphology of short nanorods or nanoflakes depending on the synthesis conditions [52]. Sample ZC100 is presented in Figure 5i,j and it is formed of bipyramidal morphology. M. Quirino et al. prepared CuO with a microwave hydrothermal method without any surfactants, and it resulted in a plate-like shape [53]. P. Gao et al. prepared CuO with a hydrothermal method without any surfactants, and it resulted in a dumbbell-like morphology [54]. H. Chen et al. prepared CuO with a hydrothermal method in the presence of sodium dodecyl benzenesulfonate as a soft template, which resulted in the formation of 3D nanobundles consisted of nanorods [55]. *Acacia nilotica* phytoexctract acted as capping agents which enhances the growth of CuO bipyramid.

Adding 25% copper oxide into zinc oxide resulted in changing the morphology from stacked layers/aggolmerated particles into homogenous elongated particles, as can be depicted in Figure 5c,d. Increasing copper oxide to 50% resulted in intermeshed flakes of ZnO along with CuO particles, Figure 5e,f. In sample ZC75, Figure 5g,h, the zinc oxide is represented by rosette shape and copper oxide has a smaller particle size compared to sample ZC50. H. Ullah et al. prepared ZnO-CuO nanocomposites with vegetable waste extract of cauliflowers, potatoes, and peas, and they found that the extract type has an impact on the morphology. The morphology of ZnO-CuO prepared with cauliflowers, potatoes, and peas were rods, a mixture of rods/spindles, and nanoparticles, respectively [56]. V. Kumari et al. prepared ZnO-CuO with a hydrothermal synthesis without any surfactants with a Zn/Cu molar ratio = 8:1, which resulted in a bittergourd morphology [57]. M. Mansournia et al. reported that CuO did not form individual morphology of CuO when CuO = 0.4, 2 and 10% CuO. However, CuO nanoplates were formed when the CuO increased to 50% along with ZnO-CuO nanoparticles [51]. P. Lu et al. prepared CuO-ZnO nanocomposites via hydrothermal method and reported that pristine CuO and pristine ZnO have nanosheets that collapsed into smaller sheets with increasing CuO [58]. T. Chang et al. prepared CuO-ZnO nanocomposites via hydrothermal method and reported that the morphology of pristine ZnO is nanoplates and the morphology of CuO in ZnO-CuO nanocomposites is nanoparticles well-distributed with ZnO [59].

**Figure 6:**
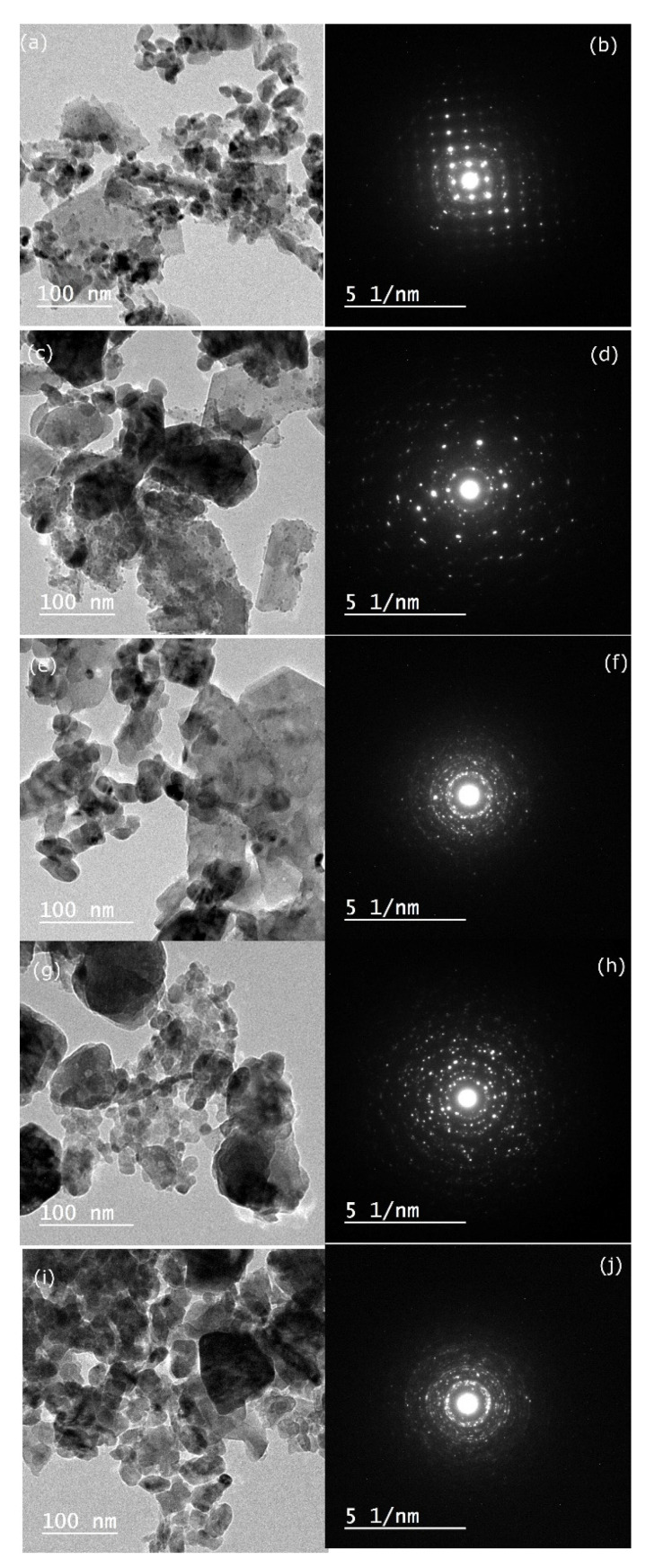
The TEM photos of ZC0 (**a**,**b**), ZC25 (**c**,**d**), ZC50 (**e**,**f**), ZC75 (**g**,**h**), and ZC100 (**i**,**j**) samples.

Figure 6 represents the TEM photos for the samples after calcination. The SAED images of all the samples approve the formation of crystalline materials, which is reflected through concentric rings which have been discussed in the XRD results. The average particle size drawn from TEM is about 13 nm, 14 nm, 15 nm, 26 nm, and 27 nm for samples ZC0, ZC25, ZC50, ZC75, and ZC100, respectively. Figure 6a,b represents sample ZC0 and its images show spherical particles and polygonal shapes. Figure 6c,d, sample ZC25, shows a ZnO elongated shape on the bottom left corner with small spherical particles on its surface, which may be referred to as CuO. Sample ZC50, in Figure 6e,f, and Sample ZC75, in Figure 6g,h, show spherical particles of CuO along with flake of ZnO, which highlights the efficiency of the extract in heterojunction formation [60]. The same observation was reported by J. Singh et al., who prepared CuO decorated ZnO nanoflakes by a hydrothermal method assisted with cetyltetra amine bromide (CTAB). Sample ZC100 shows spherical particles of CuO, as shown in Figure 6. The TEM results are in agreement with SEM results.

In the present study, ZnO NPs, ZnO-CuO NCs, and CuO NPs have been prepared by an inexpensive and environmentally friendly method; the green-hydrothermal method. The proposed mechanism is presented in the following equations;

CuCl_2_.6H_2_O (aq) + 2KOH + phytoconstituents of extract → Cu(OH)_2_ capped with phytoconstituents + 2KCl

ZnCl_2_ (aq) + 2KOH + phytoconstituents of extract → Zn(OH)_2_ capped with phytoconstituents + 2KCl

Cu(OH)_2_/Zn(OH)_2_ (hydrothermal treatment) → CuO/ZnO

The presence of phytoconstituents, i.e., flavonoids, tannins, and terpenoids; in the *Acacia nilotica* pods’ extract act as capping agents. The capping agents play an important role in decreasing the agglomeration and in controlling the growth during the hydrothermal step.

## 4. Conclusions

In the recent work, successful combination of *Acacia nilotica* pods’ extract with the hydrothermal method resulted in the synthesis of ZnO NPs, ZnO-CuO NCs, and CuO NPs. The prepared ZnO NPs have a hexagonal wurtzite phase and the CuO NPs have a monoclinic tenorite phase. Interestingly, ZnO-CuO NCs have a hexagonal phase for ZnO and a monoclinic phase for CuO with no secondary phases. The average crystallite size of NPs and NCs is in the range of 17–30 nm. The morphology of the particles depend on the ratio ZnO-CuO and the prepared particles showed different morphologies as agglomerated particles, nanorods, intermeshed flakes together with irregularly shaped particles.

## Figures and Tables

**Table 1:** Sample codes and corresponding volumes of precursors (mL).

Sample Code	ZC0	ZC25	ZC50	ZC75	ZC100
ZnCl_2_	100	75	50	25	0
CuCl_2_.6H_2_O	-	25	50	75	100

## Data Availability

Data only available upon request from the corresponding author.
